# Longitudinal pain trajectories and social context in fibromyalgia

**DOI:** 10.1097/PR9.0000000000001434

**Published:** 2026-04-09

**Authors:** Benjamin Mosch, Martin Diers

**Affiliations:** Clinical and Experimental Behavioral Medicine, Department of Psychosomatic Medicine and Psychotherapy, LWL University Hospital, Ruhr University Bochum, Bochum, Germany

We thank Dr. Pontes-Silva for his thoughtful and constructive commentary on our article “Long-term examination of pain and health-related outcomes in people with fibromyalgia before, during, and after COVID-19”^3^ and appreciate the opportunity to further clarify the conceptual framing of our work and to reflect on the role of social dimensions in fibromyalgia research.

We fully agree that the social domain constitutes a central component of health and functioning, as conceptualized in the World Health Organization's International Classification of Functioning, Disability, and Health (ICF).^2^ The perspective outlined by Dr. Pontes-Silva provides an important and timely reminder of the need for a more explicit and comprehensive integration of social determinants in chronic pain research.

As correctly noted, the primary aim of our study was not to conduct a socially oriented or ICF-based analysis, but to examine the long-term longitudinal stability of pain-related outcomes and their psychosocial correlates across 3 measurement points spanning the prepandemic, pandemic, and postpandemic periods. Within this framework, social variables were operationalized using established subscales of the West Haven–Yale Multidimensional Pain Inventory, which capture aspects of social support and social activity at the individual level. We acknowledge that these measures reflect only a limited segment of the broader social domain as conceptualized by ICF frameworks.^5^

Our baseline assessment was originally conducted in the context of a genetic association study on microRNA-related genetic variations in fibromyalgia,^1^ in which psychosocial and social factors were not the primary focus. Consequently, the selection of instruments at baseline prioritized feasibility and breadth over an in-depth assessment of specific psychosocial or social subdomains. This decision was guided not only by the exploratory nature of the study but also by the need to limit participant burden, particularly given the chronic symptom load and fatigue commonly experienced by individuals with fibromyalgia. Administering an extensive and highly differentiated questionnaire battery was therefore considered neither feasible nor ethically appropriate.

For the 2 follow-up assessments,^3,4^ methodological consistency was essential to ensure comparability of domains over time. As a result, we were bound to the questionnaire package used at baseline and could not retrospectively expand or refine the assessment of social variables without compromising longitudinal validity. Accordingly, our current study was conceived as a broad exploratory approach to examining long-term pain trajectories and associated health-related domains across an extended observation period.

Importantly, our findings, particularly the predictive role of social activity levels for pain severity at the most recent assessment, underscore the clinical relevance of social engagement, even when assessed in a relatively narrow form. We view this result as complementary to, rather than in conflict with, broader sociostructural and relational models of pain. Indeed, our interpretation explicitly cautions against unilateral causal assumptions and highlights the reciprocal relationship between pain and social participation.

Dr. Pontes-Silva also raises a point regarding gendered social roles and cumulative social burden in fibromyalgia, particularly in light of our all-female sample. While these dimensions were beyond the scope of the present analysis, we agree that they represent relevant social determinants that warrant consideration in future research.

We also concur that future longitudinal studies would benefit from a more comprehensive and theoretically explicit integration of social variables, including structural (eg, employment conditions, socioeconomic security), relational (eg, invalidation, quality of interpersonal relationships), and participatory dimensions. Such approaches would undoubtedly enrich the biopsychosocial understanding of fibromyalgia and strengthen the interpretability, external validity, and clinical translation of research findings.

In this sense, we see Dr. Pontes-Silva's letter not as a critique of our methodological choices, but as a valuable contribution to an ongoing conceptual discussion within the field. We believe that our longitudinal findings can serve as a foundation for future research that more explicitly bridges psychological processes with broader social and environmental determinants of pain and disability in fibromyalgia.

We thank the author and the editor for fostering this constructive exchange and for situating our study within a broader conceptual reflection on how the social domain is represented in contemporary fibromyalgia research. We believe that this exchange will encourage continued methodological and theoretical development in the field.

## Disclosures

The authors have no conflicts of interest to declare.
